# Psychometric properties of the 26-item eating attitudes test (EAT-26): an application of rasch analysis

**DOI:** 10.1186/s40337-022-00580-3

**Published:** 2022-05-04

**Authors:** Natalie M. Papini, Myungjin Jung, Amanda Cook, Nanette V. Lopez, Lauren T. Ptomey, Stephen D. Herrmann, Minsoo Kang

**Affiliations:** 1 Department of Health Sciences, Northern Arizona University, 1100 S. Beaver St., Flagstaff, AZ 86001 USA; 2 Department of Health, Exercise Science, and Recreation Management, The University of Mississippi, University, MS USA; 3 Volunteer Behavioral Health Care System, Murfreesboro, TN USA; 4 Department of Internal Medicine, University of Kansas Medical Center, Kansas City, KS USA; 5 Sanford Research, Sanford Health, Sioux Falls, SD USA

**Keywords:** Eating attitudes test, Rasch analysis, Psychometrics, Eating disorders

## Abstract

**Background:**

The 26-item Eating Attitudes Test (EAT-26) is a commonly used tool to assess eating disorder risk. The purpose of this study was to examine the psychometric properties of the EAT-26 with a combined sample: (1) of adults with overweight and obesity enrolled in a behavioral weight loss program and (2) general adult sample (n = 469; age = 36.17 ± 17.83 years; female = 72.5%; white = 66.3%; obese BMI category = 58%).

**Methods:**

Rasch modeling was used to assess model-data fit, create an item-person map to evaluate relative distribution items and persons, item difficulty, and person’s eating disorder (ED) risk level of the EAT-26. Differential item functioning (DIF) and rating scale functioning of the EAT-26 were also evaluated using Rasch analysis.

**Results:**

A total of 7 misfit items were removed from the final analysis due to unacceptable Infit and Outfit mean square residual values. The item-person map showed that the items were biased toward participants with moderate to high levels of ED risk and did not cover those who had low risk for having an ED (< − 1 logits). The DIF analyses results showed that none of the items functioned differently across sex, but 5 items were flagged based on obesity status. The six-category Likert-type rating scale did not function well indicating a different response format may be needed.

**Conclusion:**

Several concerns were identified with the psychometric evaluation of the EAT-26 that may question its utility in assessing ED risk in individuals at low risk for ED, within samples of people who have overweight and obesity seeking weight loss treatment.

**Plain English Summary:**

The 26-item Eating Attitudes Test is a self-rated measure of eating attitudes that measures symptoms and concerns of eating disorders (ED). Very little is known about how this instrument performs differently based on individual factors like body mass index (BMI) and sex (male/female). We used an advanced measurement theory (i.e., Rasch analysis) to determine if the EAT-26 is an adequate measure to detect disordered eating in men and women of different BMIs. Results indicated that the EAT-26 was biased toward participants with moderate to high levels of disordered eating risk and did not adequately detect individuals at low risk for disordered eating. The EAT-26 did not function differently based on sex (male/female). However, five questions did function differently based on obesity status (those without obesity/ those with obesity). Finally, we observed the six-category rating scale did not function appropriately and that a new response format may be warranted. In sum, there were several issues (e.g., poor rating scale and different item functioning) with the EAT-26 and future work should develop screening tools that detect low risk of disordered eating as well as function well in adults with overweight and obesity.

**Supplementary Information:**

The online version contains supplementary material available at 10.1186/s40337-022-00580-3.

## Background

Research indicates an increase in point prevalence of eating disorders (EDs) over recent years from 3.5% in 2000–2006 to 7.8% in 2013–2018 [1]. After substance use disorders, EDs (primarily, anorexia nervosa [AN]) have the second highest mortality rate of any mental health condition [2]. These findings highlight EDs as a public health problem and justify additional research into the prevention, screening/diagnosis, and treatment of EDs. One screening tool commonly used to detect EDs in both clinical and non-clinical populations is the 26-item Eating Attitudes Test (EAT-26) [3, 4], a scaled down version from the original 40-item instrument [5]. While this tool was originally developed as a 40-item measure to detect AN, later research found that the EAT was able to detect bulimia nervosa (BN) and other types of disordered eating [6, 7].

As one of the most widely used self-report measures for symptoms and behaviors associated with EDs, the EAT-26 has been used extensively and is recommended for use in both clinical and non-clinical settings. In its initial use, a score of 20 or greater indicated further diagnostic investigation from a qualified professional. The EAT-26 includes a three-factor structure. The “Dieting” factor contains 13 items and is characterized by scrutiny of calorie content, carbohydrates, and sugar content that is motivated by a desire to be thinner. The “Bulimia and food preoccupation” factor includes 6 items and is described by the tendency to purge after meals as well as excessive food-related thinking. The remaining seven items belong to the “Oral control” factor which reflects the tendency toward self-control of eating [5]. Over time, the EAT-26 factor structure has changed to include a three factor, four factor, five factor, and seven factor structure [8–16]. Failure to replicate the factor structure of the EAT-26 may be due to the use of the EAT-26 within samples different from the one it was originally developed (a clinical sample of females with AN) [17].

Originally, the eating attitudes test was developed and studied with clinical/diagnosed samples comprising adolescent females [3]. The use of the EAT-26 has since expanded to include individuals with overweight and obesity, individuals from different cultural backgrounds and ethnicities, and men [18–21]. Several studies leveraged the EAT-26 as a screening instrument for clinical trials aimed at adults with overweight and obesity, despite little evidence supporting the use of this tool on identifying disordered eating tendencies in this sample [22–24]. After stratifying university student participants based on normal weight and overweight/obese status, Desai et al. [25] found that a score of 11 or greater on the EAT-26 was associated with overweight. Individuals in the overweight and obese group reported higher levels of fear of binging, preoccupation with food, desire to be thinner, and dieting behavior than those in the normal weight group [25]. An EAT-26 score of 11 demonstrated better sensitivity and specificity for identifying BN, binge eating disorder (BED), and eating disorder not otherwise specified (EDNOS) in participants with obesity [23]. A cut-off score of 11 is a considerable departure from the cutoff-score of 20 originally believed to be indicative of disordered eating tendencies. Additionally, results from more recent work indicate EDs and overweight/obesity co-occur with BED being the most prevalent eating disorder in populations with overweight and obesity [26]. Binge eating behaviors are most commonly reported in individuals seeking weight loss treatments, and an estimated 30% of all individuals seeking weight loss treatments show signs of BED, defined as overeating while concurrently experiencing loss of control accompanied by feeling guilt or shame after overeating without engaging in some compensatory behavior (such as exercise or induced vomiting) [27–29]. Other signs of BED include eating alone and eating rapidly. Given these findings are unique to individuals with a BMI > 25 kg/m^2^, further research is needed to better understand how the EAT-26 operates in adults in larger bodies.

Furthermore, men are underrepresented in research on EDs despite calls for person-centered EDs treatments for men and increasing rates of EDs in men [30–32]. In a critical review, Murray et al. [33] posit EDs in men are systematically overlooked, presentations of EDs in men are very different from presentations of EDs in women, and as a result the assessment and clinical practice related to men with EDs is impacted in a way that marginalizes men with EDs. A majority of the screening tools for EDs were normed and developed based on how EDs present in women [34]. Given that men enroll in weight management programs and that extreme dieting, purging, and subthreshold BED has been shown to increase at a faster rate in men than in women [35, 36], further work is needed to understand whether common screening tools, such as the EAT-26, function differently based on sex [35, 36].

Unlike classical test theory techniques where the purpose is to find a model that best fits the data, Rasch model requires the data to fit the model in order to generate objective measurement [37]. Rasch analysis permits the examination of the functioning of the response categories, the unidimensionality of the measure, and targeting (defined as the examination of the difference between the average person measure and the average item measure of the dataset) of the measure [38]. The Rasch model is preferred over traditional methods that incorporate classical test theory because it accounts for the difficulty level of individual items and transforms responses based on ordinal scales into interval scale via logits [39–41]. Finally, the Rasch model permits study of spread, redundancy, and gapping across a wide range of person ability scores through an item-person map [39, 42]. Prior work examined the 10-item Eating Attitudes Test (EAT-10) using Rasch Analysis and found significant limitations of the tool related to structural validity and internal consistency, namely a floor effect was observed as well as redundancy of items [43]. To our knowledge, no study has examined the EAT-26 using Rasch Analysis that includes a sample permitting comparison of differential item functioning between normal weight adults and adults with overweight and obesity enrolled in a clinical weight loss program. Studies are limited that examine how the EAT-26 performs in other samples, namely: nonclinical samples of adult men and women and in a variety of Body Mass Index (BMI) statuses (kg/m^2^; overweight = 25–29.9; obesity = 30+).

The purpose of this study was to assess the psychometric properties of the EAT-26 using Rasch Analysis in adult men and women from a college/university and adult men and women with overweight and obesity enrolled in a behavioral weight management program. Additionally, it is important to understand if the EAT-26 functions differently based on key demographic factors, namely: sex and BMI status. The samples utilized in the current study allow for examination of both young adults as well as middle-aged adults, include a variety of BMI levels, and include both men and women. Using modern measurement theory may provide greater insight into the utility of the EAT-26 as an indicator of disordered eating behaviors and tendencies and may also shed light on limitations involved with using the EAT-26 to screen EDs in adults with overweight and obesity. These study aims are exploratory by nature, and findings from the current study are meant to further knowledge on appropriate screening for EDs in adults with overweight and obesity.

## Methods

### Participants

This sample consisted of 469 participants (males = 129 and females = 340) with an average age of 36.17 (*SD* = 17.83) years, with the majority of participants identifying as White (66.3%). This study combined participants from two study samples (university students enrolled in a southeastern university in the United States [Group 1] and adults with overweight and obesity enrolled in a behavioral weight loss program at a midwestern university in the United States [Group 2]) into a larger sample in order to evaluate the psychometric properties of the EAT-26. Participants from group 1 were university students who were eligible to participate if they were aged 18 and older. Recruitment methods used in group 1 were convenience sampling in classrooms that represented a variety of classifications (freshmen-seniors) and disciplines (nursing, exercise science, nutrition, sociology).

Participants from group 2 were recruited through newspaper advertisements, email listservs, public service messages, media contacts, and word of mouth. Individuals were eligible to participate if they were aged 18–65 with a BMI of 25–44.9 kg/m^2^. If participants from group 2 had a chronic medical condition, they had to receive approval to participate in the behavioral weight loss intervention from their primary care provider. Finally participants in group 2 were excluded from participating in the study if they were unwilling to be randomized, participated in a research project involving physical activity or weight management in the previous 6 months, reported planned exercise that exceeded 500 cal per week, reported weight change of ± 2.27 kg for 3 months prior to study start, were pregnant in the 6 months prior to study start or were lactating or planning to become pregnant during the 18-month trial, reported a serious medical risk (i.e., cancer, recent cardiac event), exhibited disordered eating as determined by the EAT-26 (scores of 20 or greater) with physician disproval, or exhibited extreme weigh control behaviors, engaging in special diets, or did not have access to meal preparation or shopping (i.e., college students on meal plans). During screening for eligibility in group 2, two participants were excluded from participation (group 2) based on EAT-26 scores. As a result, the presence of selection bias in the current study as it pertains to the EAT-26 scores analyzed is of limited concern. Furthermore, 11 people were excluded for having a BMI < 25 kg/m^2^ or > 45 kg/m^2^. Another two individuals were excluded for medications associated with weight change. Fifteen people were excluded for reporting planned exercise that exceeded 500 cal per week, Finally, one individual was excluded from participating in the study for medical condition, one individual was in enrolled in another study, and individual had a spouse in the study. In the current study, the average BMI was 33.87 (*SD* = 11.65) and an estimated 58% (n = 272) had obesity based on BMI ≥ 30 kg/m^2^. Table 1 indicates demographic characteristics in this study.

**Table 1:** Demographic characteristics (n = 469)

Characteristics	Group 1	Group 2	*P*-value	Total
	n = 216	n = 253		N = 469
	M ± SD	M ± SD		M ± SD
Age (years)	20.60 ± 4.91	49.68 ± 13.38	< .001	36.17 ± 17.83
Gender (%)			.03	
Male	32.41	23.32		27.5
Female	67.59	76.68		72.5
Race/ethnicity (%)			.44	
White	52.78	77.87		66.31
Black	40.28	15.42		26.87
All others	6.94	6.71		5.54
BMI (kg/m^2^)	25.26 ± 6.99	41.21 ± 9.62	< .001	33.87 ± 11.65
Obesity (%)			.04	
Obesity	17.13	94.07		58.24
Non-obesity	82.87	5.93		41.76
EAT-26 Total scores	9.37 ± 7.55	13.42 ± 8.66	< .001	11.55 ± 8.40

*SD* Standard deviation

### Eating attitudes test scale

The Eating Attitudes Test (EAT-26; 3) is a self-rated measure of eating attitudes, including a 26-item scale that measures symptoms and concerns of EDs.

*Structure*. The EAT-26 consists of three sections: (a) self-reported height and weight to create a body mass index (BMI), (b) 26 items rated on a six-point Likert scale related to how often an individual engages in certain behaviors (“Always,” “Usually,” “Often,” “Sometimes,” “Rarely,” and “Never”), and (c) five behavioral items on a six-point Likert scale examining how often a person has engaged in disordered eating behaviors over the past 6 months (“Never,” “Once a month or less,” “2–3 times a month,” “Once a week,” “2–6 times a week,” and “Once a day or more”) [5, 44]. This study only examined the 26 items of the EAT-26 and did not take into account the other two sections.

*Scoring.* Responses for items 1–25 are scored on a 4-point scale with “Always” receiving three points, “Usually,” receiving two points, “Often,” receiving one point, and “Sometimes,” “Rarely,” and “Never” receiving zero points. Item 26 is reverse scored, and a final score is calculated by summing items 1–26.

*Missing data and data screening.* Given the secondary nature of this study, data were received having already been cleaned with no missing values to report.

*Validity.* Despite the inability to replicate the factor structure of the EAT-26 across different studies, some findings indicate that scores on the EAT-26 in general populations and patient samples have been shown to be highly reliable (e.g., Cronbach’s alpha = 0.91 and Pearson *r* = 0.98) and valid (e.g., criterion validity = 0.90) [4, 5, 45]. Referral to a health care provider for clinical evaluation of ED is based on a combination of BMI, a total score of 20 or higher on the 26-items, and answers to several behavioral questions regarding eating patterns and weight loss [5].

### Data analysis

The Rasch measurement statistical software, Winsteps (version 3.65), was carried out to perform Rasch analysis. A two-facet Rasch model was estimated, including the item difficulty (difficulty level of EAT-26 items) and person ability (individual scores of EDs) parameters in logits. The Rasch model was defined with the following formula:

$$Ln\left[ {P_{njk} /1 - P_{nj(k - 1)} } \right] = D_{n} {-}C_{j} {-}F_{k}$$

where *Pnjk* is the probability of an EAT-26 item *n* being endorsed *k* category by person *j*; *Pnj*(*k*–1) is the probability of an EAT-26 item *n* being endorsed *k–*1 category by person *j*; *Dn* is the difficulty level of the EAT-26 item *n*; *Cj* is the ability level of the person *j*, and *Fk* is the threshold between category step *k* and category step *k–*1 of a scale. An item response is determined by the item difficulty (i.e., “difficulty” or severity level of eating disorder items) and person ability (i.e., the extent to which an individual may be at risk for having an eating disorder) and are expressed in logit scores. The graded response model is the IRT model, which allows for separate discrimination parameters and separate category response parameters to be estimated for each item. However, with more parameters that need to be estimated, the sample size requirements are typically larger than for a simpler model, such as Rasch model. Rasch rating scale model estimates fewer parameters and further assumes that the thresholds for category response are also equal across items. Taking into account the sample size of this study and EAT-26 items that have same category labels, the Rasch rating scale model was chosen. The following six steps are included in the Rasch calibration process.

First, model-data fit was assessed by measuring Infit and Outfit statistics in the Rasch model [46]. The Infit and Outfit statistics are the information-weighted mean square residuals between observed and expected responses, but Outfit measure is more sensitive to the outlier results. Infit and Outfit measures with a value close to 1 denote an adequate model-data fit. If Infit and Outfit statistics are less than 0.5 or greater than 1.5, it should be considered a poor fit [46]. Infit and Outfit values greater than 1.5 show large variability in responses and values less than 0.5 indicate little variation. The problematic items were deleted and reanalyzed until Infit and Outfit values were satisfactory. In addition, a unidimensionality of the scale and local independence of the item were evaluated based on Linacre’s guidelines [47]. The dimensionality of the scale and local independence of the item were examined by conducting Rasch factor analysis using Principal Component Analysis of the standardized residuals and a residual correlation, respectively. The unidimensionality is satisfied if the first contrast (component) is not much bigger than two eigenvalues. The items in the scale are locally independent of each other if a residual correlation is not greater than 0.7.

Second, the function of the rating scale was analyzed to determine whether the existing Likert categories (i.e., six Likert categories) were appropriate for the items. Suitable functioning was evaluated based on the following criteria: (a) regular observation distribution, (b) average logit score measures advancing with category, (c) appropriate mean square residual of outfit statistics (< 2.0), and (d) advancing category thresholds (i.e. boundaries between rating categories) [48].

Third, an item-person map distribution was examined. The map visually illustrates logit scores of both item and person on the same scale, thus allowing the comparison of these measures. It also shows both item and person distributions as well the relative position of individual level of risk for the EAT-26 items in logits.

Fourth, the parameters for item difficulty were calculated during the calibration process in logits. The higher the logit score, the more difficult it was to agree with the item/ the greater level of eating disorder symptomatology is needed for a participant to endorse the item. Item separation index and item separation reliability were also examined. The item separation index indicates how well items are separated along a measurement scale. A separation score greater than 2.0 indicates acceptable separation for items [39]. The item separation reliability shows the ability to replicate item placements along the measurement scale if these same questions were given to another sample. The item separation reliability close to 1.00 denotes a high degree of confidence for item [49].

Fifth, the individual’s level of EAT was estimated in which the higher the logit score, the higher risk of having an eating disorder. Person separation index and person separation reliability were also investigated. The person separation index indicates how well people are spread along a measurement scale. A separation score greater than 2.0 signals acceptable separation [39]. The person separation reliability indicates the reproducibility of person’s placement when they responded to another set of EAT items. The person separation reliability near 1.00 indicates a high degree of confidence for person [50].

Finally, a differential item functioning (DIF) analysis was conducted to demonstrate if items in EAT-26 function differently by sex and obesity status (BMI ≥ 30 kg/m^2^). DIF implies that the item difficulty between groups is different, but it may be biased towards a specific group and question the validity of the instrument [51]. Items were considered to be biased when they exhibited both substantive (i.e., Mantel–Haenszel [M-H] DIF size > 0.43 logits) and statistical significance (*p* < 0.001); [52]. If the M-H DIF is larger than 0.43 logits, the functions were different among the groups. The significance level of the DIF analysis was set to 0.001 to account for potential inflation at the alpha level in multiple comparisons. Data obtained in the original studies were approved by the Institutional Review Boards at Middle Tennessee State University and the University of Kansas. Written informed consent was obtained prior to participation in both studies. The activities involved in the current study was determined to not meet the definition of human subjects research (as cited in the regulations issued by the U.S. Department of Health and Human Services) by the Institutional Review Board at Northern Arizona University. Since this study involves the use of secondary data, the study team did not intervene or interact with individual participants or have access to identifiable private information.

## Results

### Model-data fit

Model-data fit was evaluated for EAT-26 items using Infit and Outfit statistics. In the initial analysis, 4 items were flagged due to the occurrence of high Infit and Outfit statistics (see Additional file 1: Table S1). Item 8 (Infit = 1.58, Outfit = 1.98), item 13 (Infit = 2.15, Outfit = 3.23), item 25 (Infit = 1.55, Outfit = 1.70), and item 26 (Infit = 1.75, Outfit = 2.24) had both Infit and Outfit statistics above the acceptable range. When a second Rasch analysis was performed without items 8, 13, 25, and 26, items 15 and 19 had unacceptable Infit values of 1.52 and 1.66 and Outfit values of 1.69 and 1.93, respectively (see Additional file 1: Table S2). When a third Rasch analysis was conducted without items 8, 13, 15, 19, 25 and 26, item 9 had unacceptable Outfit value of 2.25. Item 5 also had Outfit statistics (1.68), but this item was not removed since its’ Infit value (1.43) was within the acceptable range and the question was important to measure the eating disorder construct (see Additional file 1: Table S3). Therefore, a total of 7 items were removed from a final analysis. The resulting Infit statistics range was 0.67–1.43, and the resulting Outfit statistics range was 0.74–1.68 (see Additional file 1: Table S4). In addition, the results from Principal Component Analysis of the standardized residuals and a residual correlation showed that unidimensionality of this scale was satisfied (i.e., an eigenvalue on the first contrast was 2.3) and the items of this scale were locally independent of each other (i.e., a residual correlation was not greater than 0.7).

### Rating scale functioning

**Fig. 1:**
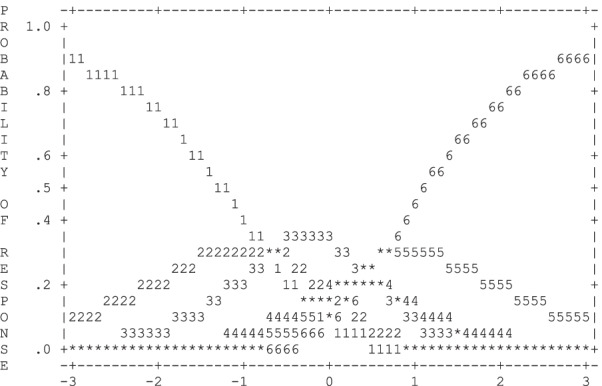
Six-point Likert response category probabilities for EAT-26 scale

A summary of how the six-category Likert rating scale of the EAT-26 functioned is provided in Table 2. Overall, the six-category rating scale did not function well. The average logit measures advanced as category increased, and the outfit statistics fell within the desired range of < 2.0. However, the category thresholds were not arranged in sequence. This is clearly illustrated in the Fig. 1 by noting there is no independent peak such as what is observed with categories 4 and 5. This disordering of thresholds values could be a signal that the rating scale may be problematic.

**Table 2:** Summary of EAT-26 scale rating scale function

Category score	Counts used	Average measure	Outfit MNSQ	Category thresholds
1 (Never)	2791	− 1.40	0.98	None
2 (Rarely)	2003	− 0.74	0.94	− 0.74
3 (Sometimes)	2063	− 0.46	1.06	− 0.66
4 (Often)	840	− 0.16	1.08	0.60
5 (Usually)	682	0.04	1.13	0.19
6 (Always)	464	0.36	1.11	0.61

Average measure = a mean of logit measures in category; MNSQ = mean square residuals

### Item-person map

**Fig. 2:**
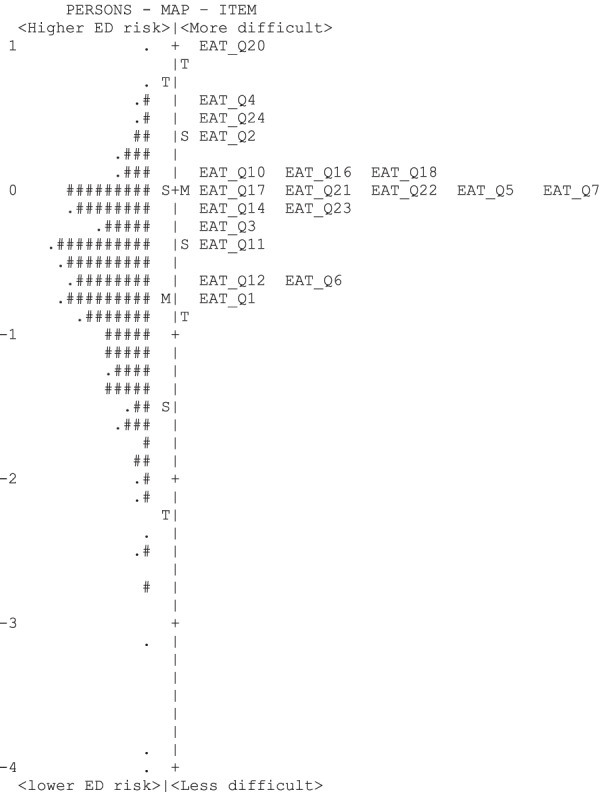
Item-person map of EAT-26 scale. *Note*: Each “M” represents a mean of person’s level of eating disorder risk on the left side and a mean of item’s difficulty on the right side. “S” and “T” represent one standard deviation and two standard deviation points, respectively

Figure 2 presents the item-person map of the EAT-26. The distribution of a person’s level of risk of EDs, which is indicated by the # symbol and dots, is illustrated on the left side of the map, whereas the distribution of the EAT-26 items is displayed on the right side based on their difficulty levels. The map shows that participants’ EAT levels were normally distributed along the logits scale. The items were clustered in an area differentiated participants with moderate to high levels of eating disordered risk and did not cover those who had low risk for having an ED (< − 1 logits). Moreover, several items (e.g., items 5, 7, 17, 21 and 22) appeared have a similar location on the logits scale (i.e., similar difficulty level). This limits their utility to differentiate individuals’ level of eating disorder risk.

### EAT-26 item difficulty

The EAT-26’s item difficulty, standard errors, and infit and outfit statistics are reported in Table 3. The higher the logits, the more difficult it was for the participants to agree with the item/the greater level of eating disorder symptomatology is needed for a participant to endorse the item. Item difficulty ranged from − 0.72 to 1.03 logits. The calibration results showed that the most difficult to agree item was “Feel that others pressure me to eat” (logit = 1.03), whereas the least difficult to agree item was “Am terrified about being overweight” (logit =  − 0.72). The item separation index was 9.39, indicating that the EAT-26 items were well distributed across the measurement scale. The separation reliability of the items was 0.99, pointing to a high degree of confidence in replicating the placement of the items within the measurement error of another sample.

**Table 3:** Item summary of Rasch calibration in EAT-26 Scale

Item	Calibration logits	SE logits	Infit MNSQ	Outfit MNSQ
Q20. Feel that others pressure me to eat	1.03	0.06	1.37	1.24
Q4. Have gone on eating binges where I feel that I may not be able to stop	0.63	0.05	1.31	1.21
Q24. Like my stomach to be empty	0.55	0.05	1.19	1.09
Q2. Avoid eating when I am hungry	0.40	0.05	0.83	0.94
Q16. Avoid foods with sugar in them	0.15	0.04	1.00	1.03
Q10. Feel extremely guilty after eating	0.15	0.04	0.75	0.67
Q18. Feel that food controls my life	0.13	0.04	1.13	1.05
Q5. Cut my food into small pieces	0.00	0.04	1.43	1.68
Q22. Feel uncomfortable after eating sweets	− 0.01	0.04	0.83	0.81
Q21. Give too much time and thought to food	− 0.02	0.04	0.98	0.93
Q17. Eat diet foods	− 0.02	0.04	0.69	0.76
Q7. Particularly avoid foods with a high carbohydrate content (i.e., bread, rice, potatoes, etc.)	− 0.06	0.04	0.97	1.02
Q23. Engage in dieting behavior	− 0.15	0.04	0.67	0.74
Q14. Am preoccupied with the thought of having fat on my body	− 0.18	0.04	1.04	1.00
Q3. Find myself preoccupied with food	− 0.20	0.04	0.94	1.07
Q11. Am preoccupied with a desire to be thinner	− 0.42	0.04	0.79	0.79
Q12. Think about burning up calories when I exercise	− 0.62	0.04	1.34	1.37
Q6. Aware of the calorie content of foods that I eat	− 0.64	0.04	1.11	1.24
Q1. Am terrified about being overweight	− 0.72	0.04	1.19	1.18

*SE* Standard errors; *MNSQ* mean square residuals

### Individual level of eating disorder

The person’s ability level, or individual level of risk of having an eating disorder, was estimated through the Rasch calibration process in logits, wherein a higher logit value corresponds to a higher risk of EDs. The average of EAT levels was -0.72 (SD = 0.77). The range of risk level of all participants was from − 5.00 to 0.99 logits, indicating a wide spread of risk. Person separation was 2.51, denoting that person’s ability are separated along the measurement continuum indicating that the EAT-26 is sensitive enough to distinguish about 3 statistically distinct strata, which is acceptable. Person separation reliability was 0.86, signifying an acceptable degree of confidence in replicating placement of persons within a measurement error.

### Differential item functioning

Differential item functioning (DIF) analyses were completed for sex and obesity status. None of the items functioned differently across sex with no items having a statistically significant (*p* < 0.001) and M-H DIF size > 0.43 logits. For DIF analysis for the obesity status subgroups, items 3, 12, 14, 18 and 20 were flagged, reflecting differences in item difficulty between people with obesity (BMI ≥ 30 kg/m^2^) and individuals without obesity. Specifically, items 12, 14 and 20 were more difficult to agree for participants with obesity (logits =  − 0.36, − 0.07 and 1.19) than participants without obesity (logits =  − 1.03, − 0.36, and 0.76), whereas participants with obesity(logits =  − 0.32 and − 0.08) were more likely to agree with items 3 and 18 than participants without obesity (logits = 0.02 and 0.64).

## Discussion

The purpose of this study was to examine the psychometric properties of the 26-item Eating Attitudes Test (EAT-26) using Rasch Analysis in adult men and women from (1) a college/university and (2) individuals with overweight and obesity enrolled a behavioral weight management program. These two samples were combined in the current study to evaluate the psychometric properties of the EAT-26 and to determine if the instrument functions differently based on subgroup affiliations.

Model data fit indicated a total of 7 misfit items which were removed from the final analysis based on unacceptable infit and outfit mean square residual values. Of the 7 items removed from analysis, the majority were in the oral control factor (items 8: “*Feel that others would prefer if I ate more*,” 13: “*Other people think that I am too thin*,” 15: “*Take longer than others to eat my meal,”* and 19: “*Display self-control around food*”). The oral control factor pertains to self-control of eating and perceived pressure from others to gain weight [5]. Item-difficulty identified item 20: “*I feel that others pressure me to eat*” as the most difficult to agree (logit = 1.03), while the least difficult item to agree was item 1: “*I am terrified about being overweight*” (logit =  − 0.72). Given the current study sample average BMI was 33.87 and an estimated 58% had obesity based on BMI ≥ 30, the majority of participants in the sample rated the fears of being overweight item consistently “always,” or “often.” It is well-established that people with overweight and obesity experience bias, discrimination, and ridicule based on body shape and size [53, 54]. Internalized weight bias, also known as weight self-stigma, occurs when an individual with overweight or obesity internalizes negative beliefs and stereotypes about people in larger bodies [55]. It is possible that fear of being overweight was rated “always” or “often” across this sample, in part, because of internalized weight bias from those with overweight and obesity. Items related to food-preoccupation (described by the tendency to experience excessive food-related thinking) showed lower difficulty level in participants with obesity. This could partially be explained by the fact that participants in group 2 were enrolled in a weight management program and may be more concerned about their weight and food intake than a person with obesity who did not enroll in a weight management program. Previous findings indicate that adults with overweight and obesity who were randomly assigned to a daily energy restriction diet (high-protein, meal replacement program) experienced greater preoccupation with food than those assigned to a daily energy restriction program with alternate day fasting [56]. Item 9 “ I vomit after I have eaten,“ item 25 “I have the impulse to vomit after meals,” and item 26 “I enjoy trying new rich foods” were also removed as a result of unacceptable infit/outfit statistics. Removal of items 9 and 25 could be a result of a known decline in bulimia nervosa incidence rate over time [57]. Furthermore, the use of such transparent items that ask about purging behaviors could elicit social desirability bias in participants where they may be less likely to endorse items that include stigmatizing behaviors [58]. Lastly, item 26 could have flagged for inadequate infit/outfit statistics given some people in the present sample were enrolled in a behavioral weight loss program. This is consistent with other findings that suggest individuals with overweight and obesity enrolled in a 12-month weight loss program had a greater tendency to avoid foods high in refined carbohydrates [59]. After removal of items 8, 9, 13, 15, 19, 25 and 26, the remaining 19 items exhibited fit appropriate to the expectations of the model.

The item-person map shows that the items differentiated participants with moderate to high levels of eating disorder risk and did not differentiate between those with low risk for having an eating disorder (< − 1 logits). This allows the positions of item difficulties and person abilities screened to be easily examined visually and to note any gaps. Identification of gaps in item distribution could be used to help guide the development of new items and remove the duplicated items. No items from the EAT-26 discriminated people at lower risk of disordered eating. It is important to incorporate screening tools that differentiate participants at all risk levels for developing an eating disorder, including those who are considered “low risk.” The use of tools that adequately identify participants at all risk levels of developing an ED is especially critical if future research were to longitudinally examine disordered eating over time. Longitudinal study of eating disorders using the EAT-26 may be inappropriate since it does not properly identify those at low risk. For example, Richardson et al. [60] examined the relationship between financial difficulties and eating attitudes in university students and observed higher eating attitudes scores at baseline significantly predicted greater financial difficulties at 3–4 months. However, in light of the current study findings, these findings may be due to the psychometric limitations of the instrument in that it did not adequately discriminate participants at lower levels of eating disorder risk and use of a different eating disorder measure would be more appropriate. Additionally, items 10: “*Feel extremely guilty after eating*,” 16: “*Avoid foods with sugar in them*,” and 18: “*Feel that food controls my life*,” all fell on the same difficulty level of the item-person map. Items 17: “*Eat diet foods*,” 21: “Give too much time and thought to food,” 22: “Feel uncomfortable after eating sweets,” 5: “*Cut my food into small pieces*,” and 7: “*Particularly avoid food with a high carbohydrate content*” all loaded on the same level of the item-person map.

DIF analysis indicated that roughly 25% of the items retained in the EAT-26 differed based on having a BMI of ≥ 30. Items 3 (“*I find myself preoccupied with food*”), 12 (“*I think about burning up calories when I exercise*”), 14 (“*I am preoccupied with the thought of having fat on my body*”), 18 (“*I feel that food controls my life*”), and 20 (“*I feel that others pressure me to eat*”) functioned differently as a result of obesity status (obese vs. non-obese). Despite having the same risk for disordered eating, items 12, 14, and 20 were more difficult for individuals with obesity to rate “always” or “usually” than individuals without obesity. Additionally, individuals with obesity were more likely to agree with items 3 and 18 than individuals without obesity. These findings are consistent with the literature on obesity, namely that preoccupations with body and food can serve as predictors to disordered eating and chronic dieting [61, 62]. Furthermore, dieters have shown to report higher food and dieting-related thoughts than non-dieters [63]. The tendency of food preoccupation and the belief that food controls life within individuals with obesity is similar to findings reported in Desai et al. [25], namely that participants with overweight were more likely than their normal weight peers to be preoccupied with food. Findings in the present study are somewhat aligned with other work that utilized DIF to examine item bias by BMI status on the 8-item Eating Attitudes Test (EAT-8). One item on the EAT-8 functioned differently by BMI: “I am preoccupied with a desire to be thinner,” where individuals with higher BMIs reported greater preoccupation with thinness [64]. This item was not shown to differ by obesity status in the present study. It is plausible that this discrepancy could come from differences in BMI categorization. Thielemann et al. [64] recruited a large enough sample to examine DIF across all BMI categories (underweight, normal weight, overweight, and obesity) while the present study was powered to examine only those with obesity and those without obesity (dichotomous).

No items functioned differently based on sex. This finding is promising given the ED treatment issues pertinent to men, which includes: stereotypes of EDs as a “woman’s issue,” muscularity-oriented disordered eating as distinct from how EDs present in female populations, and inadequate health literacy among health practitioners in the field of EDs [32, 33, 65]. Schaefer et al. [21] reported no evidence of clinically significant differential item functioning in the EAT-26 in undergraduate men and women. Unlike the present findings related to differential functioning and sex with the EAT-26, the 12-item short form of the Eating Disorder Examination Questionnaire (EDE-QS) was examined using a Rasch analysis and differential item functioning was observed across sex groups [66]. Additionally, Thielemann et al. [64] found DIF by gender with respect to item 1 “I eat diet foods,” 4 “I feel uncomfortable after eating sweets,” and 6 “I am terrified about being overweight” on the EAT-8 [64]. It is imperative to continue to assess sex differential functioning for EDs in other eating disorder instruments since EDs in men are underdiagnosed and many men fail to recognize disordered patterns of behavior because of cultural influences of EDs as a woman’s illness [67, 68].

As shown in Table 2, the six-category Likert-type rating scale did not function well. Thresholds did not advance in order with category 1 = none, category 2 =  − 0.74, category 3 =  − 0.66, category 4 = 0.60, category 5 = 0.19, category 6 = 0.61. This indicates that a different response format is warranted. In the current study, the “Never” response option was selected 31.56% of the time, “Rarely” was selected 22.65% of the time, “Sometimes” was selected 23.33% of the time, “Often” was selected 9.5% of the time, “Usually” was selected 7.71% of the time, and “Always” was selected 5.25% of the time. Of the six-categories, there are several infrequently selected (“Always,” “Usually,” and “Often”) and one category used more frequently than other (“Never”). These findings are consistent with other work that found lower endorsements on other ED self-report measures, particularly for restrained eating-items for people with overweight and obesity [69]. Lower endorsements of Likert responses “Always,” “Often,” and “Usually” in a nonclinical sample is not unusual when the EAT-26 is applied in a non-clinical sample. It is somewhat expected that the distribution of scores on the EAT-26 in a non-clinical sample would be positively skewed, as seen in the present study. The problems inherent in the six-category Likert-type rating scale functioning of the EAT-26 may partially explain why previous studies have observed insufficient sensitivity to detect a full or partial ED and why use of the EAT-26 within samples with overweight recommend a cut-off score of 11 instead of the originally proposed cut-off score of 20 [25, 70].

Strengths of this study include an adequate sample that consists of both adult men and women and participants from different BMI categories. Additionally, the utilization of Rasch analysis overcomes several limitations of traditional methods based on classical test theory. The current study contributes to what is known about EDs, particularly in identifying differences in the EAT-26 measure amongst people with obesity. Finally, the generalizability of these findings is appropriate for others who may want to incorporate an ED questionnaire into a program or clinical trial to screen for participant eligibility or monitor disordered eating development for those enrolled in a weight-focused intervention. Because our sample includes college students and adults enrolled in a behavioral weight loss program, these findings are applicable to those working in obesity and weight loss interventions.

There are several limitations of this study. First, the EAT-26 yields a referral index that is based on three criteria: (1) the total score of the EAT-26, (2) participant responses to behavioral questions around eating symptoms and weight loss, and (3) the individual’s BMI. This study only examined the 26 items of the EAT-26 and did not take into account the other two criteria for referring respondents to a qualified professional. Previous findings show higher BMI is positively correlated with EAT-26 scores [71]. These findings contrast the recommendation of the tool using BMI, which encourages professional evaluation for a possible eating disorder if a person falls in the “extremely underweight” category compared to age-matched population norms (eat-26.com). Future work should compare differences in behavioral questions of the EAT-26 to determine if there are any problematic differences between people of different BMIs. For example, if the behavioral questions do not detect disordered eating in people at elevated BMIs because of the way the questions are designed and not because of the individual’s disordered eating behaviors, then this would further add support for the development of a separate tool that assesses disordered eating in people in larger bodies.

Social desirability is a possibility when it comes to self-report measures, especially those on sensitive topics such as EDs. It is possible participants may have responded to questions on the EAT-26 in order to present themselves in a positive light. Individuals in the sample of individuals with overweight and obesity seeking weight loss treatment may have answered in a way that indicates less ED severity in order to receive treatment. This has been observed in other work, particularly that social desirability affects the assessment of eating behavior [72]. Future work should investigate the psychometric quality of eating disorder instruments and include a measure to account for social desirability, such as the Marlow-Crowne Social Desirability Scale [73]. The EAT-26 was included as an eligibility screening tool for a behavioral weight loss intervention for some adults in the current sample. It is possible that participants were aware of eligibility requirements specified in the informed consent and failed to report disordered eating patterns within the EAT-26 with the intention of enrolling in the program. Chandler and Paolacci [74] noted the pitfalls of relying on participant self-report to determine eligibility and show participants can misrepresent relevant study inclusion criterion. The current study utilized two samples from previous studies to evaluate the EAT-26 across a variety of participants who represent a variety of demographic variables. Neither of the previous studies incorporated any additional measure of eating disorder severity or clinical diagnoses to confirm the likelihood of an ED or asked participants about diagnostic status. Concurrent or convergent validity evidences are ideal, and we recognize this as a limitation in the present study. Another limitation of this study is the lack of confirmed cases of EDs in this sample. If the current study had a record of diagnosed EDs, this would allow study of the efficacy of the EAT-26 to detect ED risk. Additionally, the Rasch analysis could be used to understand differential functioning of the EAT-26 based on eating disorder type (e.g., AN, BN, and binge eating).

As previously noted in the methods, the current study combined a sample of university students with adults enrolled in a behavioral weight loss program. Eligibility criteria were different for the two studies, where university students were required to be 18 and older while those enrolled in the behavioral weight loss program had more stringent inclusion and exclusion criteria based on previous health history and chronic medical conditions. For adults enrolled in the behavioral weight loss program (group 2), one exclusionary criterion was disordered eating based on EAT-26 scores. However, only two prospective participants were excluded from participation (group 2) based on EAT-26 scores in the original study. As a result, it is not believed the current study is reflective of selection bias as it pertains to the EAT-26 scores available and analyzed.

## Conclusions

In conclusion, use of the EAT-26 to screen for EDs is cautioned based on the findings of the current study and the significant limitations of the EAT-26. Findings in the current study can only be generalized to those individuals with overweight and obesity who opted to participate in a behavioral weight management program, and are not representative of other individuals with overweight and obesity, such as those who may opt for bariatric surgery or other forms of weight control as well as those who do not opt for any weight control method. Future work should investigate whether the EAT-26 performs adequately in other samples of individuals with overweight and obesity who select other methods of weight control (bariatric surgery or other weight management programs) and those who do not seek weight control methods (nondieters). Participants with the same level of disordered eating responded differently to certain eating disorder items on the EAT-26 as a result of differences in weight. Historically, EDs have been thought to only impact “skinny, white, affluent girls” (SWAG stereotype) [75]. However, findings from young adults illustrate that individuals with overweight and obesity are among the highest at risk of developing eating disorder symptoms, while those who fall in underweight BMI categories report the lowest risk [76, 77]. This study addresses the need to psychometrically evaluate measures of disordered eating in populations of people with obesity [78]. Findings from the present study confirm that the EAT-26 is not a good tool to use for the purpose of screening for low-risk EDs in a weight-loss treatment seeking sample of people with BMIs in the overweight and obese range. Future studies that screen for disordered eating in adults with overweight and obesity should rely primarily on interview assessments to detect binge eating behaviors and self-report measures to provide additional context on severity and shape and weight-related concerns [78]. Additionally, the use of an instrument that better detects EDs in people at higher BMIs could help surveillance in community-based programs that use dietary restriction to address overweight or obesity. Given the EAT-26 was designed to screen for AN and not specifically designed to identify the risk of EDs in people with higher body weights, a more appropriate screening tool should be adopted for the purpose of screening for risk of ED (including those at low risk for ED) in people with higher body weights, especially when applied to those seeking weight-loss treatment.

## Supplementary Information

**Additional file 1.** **Supplementary Table 1.** Initial analysis of model-data fit in EAT-26 Scale. **Supplementary Table 2.** Second analysis of model-data fit in EAT-26 Scale. **Supplementary Table 3.** Third analysis of model-data fit in EAT-26 Scale. **Supplementary Table 4.** Final analysis of model-data fit in EAT-26 Scale.

## Data Availability

The datasets used and/or analyzed during the current study are available from the corresponding author on reasonable request.

## Abbreviations

AN: Anorexia Nervosa
BED: Binge eating disorder
BMI: Body Mass Index
BN: Bulimia Nervosa
DIF: Differential item function
EAT-26: Eating attitudes test (26 item)
EDs: Eating disorders
SWAG Stereotype: Skinny, white, affluent, girls
