# Supplementary material for: Psychometric properties of the 26-item eating attitudes test (EAT-26): an application of rasch analysis
Source: J Eat Disord. 2022 May 4;10:62. doi: 10.1186/s40337-022-00580-3 (PMC9069796; doi:10.1186/s40337-022-00580-3)
Supplement: Supplementary file 1 — Additional file 1. Supplementary Table 1. Initial analysis of model-data fit in EAT-26 Scale. Supplementary Table 2. Second analysis of model-data fit in EAT-26 Scale. Supplementary Table 3. Third analysis of model-data fit in EAT-26 Scale. Supplementary Table 4. Final analysis of model-data fit in EAT-26 Scale. [file 40337_2022_580_MOESM1_ESM.docx]

| Supplementary Table 1. Initial analysis of model-data fit in EAT-26 Scale | | | | |
| --- | --- | --- | --- | --- |
| *Item* | *Calibration*  *logits* | *SE*  *logits* | *Infit*  *MNSQ* | *Outfit*  *MNSQ* |
| *Q13. Other people think that I am too thin | 0.97 | 0.06 | 2.15 | 3.23 |
| *Q26. Enjoy trying new rich food | -1.55 | 0.04 | 1.75 | 2.24 |
| *Q8. Feel that others would prefer if I ate more | 0.80 | 0.06 | 1.58 | 1.98 |
| Q9. Vomit after I have eaten | 2.01 | 0.12 | 1.29 | 1.83 |
| *Q25. Have the impulse to vomit after meals | -0.05 | 0.04 | 1.55 | 1.70 |
| Q19. Display self-control around food | -0.67 | 0.03 | 1.43 | 1.65 |
| Q15. Take longer than others to eat my meals | -0.02 | 0.04 | 1.34 | 1.44 |
| Q20. Feel that others pressure me to eat | 0.79 | 0.06 | 1.19 | 0.98 |
| Q4. Have gone on eating binges where I feel that I may not be  able to stop | 0.44 | 0.05 | 1.17 | 1.09 |
| Q5. Cut my food into small pieces | -0.08 | 0.04 | 1.08 | 1.13 |
| Q12. Think about burning up calories when I exercise | -0.59 | 0.03 | 1.09 | 1.10 |
| Q1. Am terrified about being overweight | -0.67 | 0.03 | 1.03 | 1.00 |
| Q18. Feel that food controls my life | 0.03 | 0.04 | 1.02 | 0.96 |
| Q24. Like my stomach to be empty | 0.37 | 0.05 | 1.02 | 0.95 |
| Q14. Am preoccupied with the thought of having fat on my body | -0.23 | 0.04 | 0.93 | 0.88 |
| Q6. Aware of the calorie content of foods that I eat | -0.60 | 0.03 | 0.87 | 0.90 |
| Q21. Give too much time and thought to food | -0.10 | 0.04 | 0.88 | 0.83 |
| Q16. Avoid foods with sugar in them | 0.04 | 0.04 | 0.81 | 0.83 |
| Q3. Find myself preoccupied with food | -0.25 | 0.04 | 0.78 | 0.81 |
| Q7. Particularly avoid foods with a high carbohydrate content  (i.e., bread, rice, potatoes, etc.) | -0.13 | 0.04 | 0.78 | 0.81 |
| Q22. Feel uncomfortable after eating sweets | -0.09 | 0.04 | 0.75 | 0.72 |
| Q11. Am preoccupied with a desire to be thinner | -0.43 | 0.03 | 0.75 | 0.74 |
| Q10. Feel extremely guilty after eating | 0.04 | 0.04 | 0.73 | 0.67 |
| Q2. Avoid eating when I am hungry | 0.25 | 0.04 | 0.67 | 0.71 |
| Q23. Engage in dieting behavior | -0.21 | 0.04 | 0.57 | 0.59 |
| Q17. Eat diet foods | -0.10 | 0.04 | 0.56 | 0.58 |
| Note. SE = standard errors; MNSQ = mean square residuals. *Items were removed since their values were outside the acceptable range of both Infit and Outfit statistics. | | | | |

| Supplementary Table 2. Second analysis of model-data fit in EAT-26 Scale | | | | |
| --- | --- | --- | --- | --- |
| *Item* | *Calibration*  *logits* | *SE*  *logits* | *Infit*  *MNSQ* | *Outfit*  *MNSQ* |
| Q9. Vomit after I have eaten | 2.18 | 0.12 | 1.33 | 2.19 |
| *Q19. Display self-control around food | -0.74 | 0.04 | 1.66 | 1.93 |
| *Q15. Take longer than others to eat my meals | -0.01 | 0.04 | 1.52 | 1.69 |
| Q4. Have gone on eating binges where I feel that I may not be  able to stop | 0.51 | 0.05 | 1.31 | 1.22 |
| Q20. Feel that others pressure me to eat | 0.89 | 0.06 | 1.29 | 1.12 |
| Q5. Cut my food into small pieces | -0.07 | 0.04 | 1.22 | 1.29 |
| Q12. Think about burning up calories when I exercise | -0.65 | 0.04 | 1.16 | 1.17 |
| Q18. Feel that food controls my life | 0.05 | 0.04 | 1.14 | 1.06 |
| Q24. Like my stomach to be empty | 0.43 | 0.05 | 1.10 | 1.01 |
| Q1. Am terrified about being overweight | -0.74 | 0.04 | 1.10 | 1.08 |
| Q6. Aware of the calorie content of foods that I eat | -0.66 | 0.04 | 0.99 | 1.07 |
| Q3. Find myself preoccupied with food | -0.27 | 0.04 | 0.93 | 0.99 |
| Q14. Am preoccupied with the thought of having fat on my body | -0.24 | 0.04 | 0.99 | 0.93 |
| Q21. Give too much time and thought to food | -0.09 | 0.04 | 0.99 | 0.93 |
| Q7. Particularly avoid foods with a high carbohydrate content  (i.e., bread, rice, potatoes, etc.) | -0.13 | 0.04 | 0.89 | 0.93 |
| Q16. Avoid foods with sugar in them | 0.06 | 0.04 | 0.90 | 0.90 |
| Q2. Avoid eating when I am hungry | 0.29 | 0.05 | 0.75 | 0.87 |
| Q22. Feel uncomfortable after eating sweets | -0.08 | 0.04 | 0.82 | 0.80 |
| Q11. Am preoccupied with a desire to be thinner | -0.47 | 0.04 | 0.80 | 0.79 |
| Q10. Feel extremely guilty after eating | 0.06 | 0.04 | 0.79 | 0.72 |
| Q23. Engage in dieting behavior | -0.22 | 0.04 | 0.63 | 0.68 |
| Q17. Eat diet foods | -0.09 | 0.04 | 0.62 | 0.67 |
| Note. SE = standard errors; MNSQ = mean square residuals. *Items were removed since their values were outside the acceptable range of both Infit and Outfit statistics. | | | | |

| Supplementary Table 3. Third analysis of model-data fit in EAT-26 Scale | | | | |
| --- | --- | --- | --- | --- |
| *Item* | *Calibration*  *logits* | *SE*  *logits* | *Infit*  *MNSQ* | *Outfit*  *MNSQ* |
| *Q9. Vomit after I have eaten | 2.23 | 0.12 | 1.35 | 2.25 |
| Q5. Cut my food into small pieces | -0.11 | 0.04 | 1.43 | 1.67 |
| Q12. Think about burning up calories when I exercise | -0.74 | 0.04 | 1.33 | 1.37 |
| Q20. Feel that others pressure me to eat | 0.91 | 0.06 | 1.36 | 1.24 |
| Q4. Have gone on eating binges where I feel that I may not be  able to stop | 0.51 | 0.05 | 1.30 | 1.20 |
| Q6. Aware of the calorie content of foods that I eat | -0.75 | 0.04 | 1.11 | 1.22 |
| Q1. Am terrified about being overweight | -0.84 | 0.04 | 1.18 | 1.09 |
| Q24. Like my stomach to be empty | 0.43 | 0.05 | 1.10 | 1.01 |
| Q18. Feel that food controls my life | 0.02 | 0.04 | 1.12 | 1.05 |
| Q3. Find myself preoccupied with food | -0.32 | 0.04 | 0.94 | 1.06 |
| Q14. Am preoccupied with the thought of having fat on my body | -0.30 | 0.04 | 1.04 | 0.99 |
| Q16. Avoid foods with sugar in them | 0.03 | 0.04 | 1.00 | 1.03 |
| Q7. Particularly avoid foods with a high carbohydrate content  (i.e., bread, rice, potatoes, etc.) | -0.17 | 0.04 | 0.97 | 1.01 |
| Q21. Give too much time and thought to food | -0.13 | 0.04 | 0.98 | 0.93 |
| Q2. Avoid eating when I am hungry | 0.28 | 0.05 | 0.82 | 0.94 |
| Q22. Feel uncomfortable after eating sweets | -0.12 | 0.04 | 0.83 | 0.81 |
| Q11. Am preoccupied with a desire to be thinner | -0.54 | 0.04 | 0.79 | 0.79 |
| Q17. Eat diet foods | -0.14 | 0.04 | 0.69 | 0.76 |
| Q10. Feel extremely guilty after eating | 0.03 | 0.04 | 0.75 | 0.67 |
| Q23. Engage in dieting behavior | -0.27 | 0.04 | 0.68 | 0.74 |
| Note. SE = standard errors; MNSQ = mean square residuals. *Item was removed since its value was outside the acceptable range of Outfit statistics. | | | | |

| Supplementary Table 4. Final analysis of model-data fit in EAT-26 Scale | | | | |
| --- | --- | --- | --- | --- |
| *Item* | *Calibration*  *logits* | *SE*  *logits* | *Infit*  *MNSQ* | *Outfit*  *MNSQ* |
| Q5. Cut my food into small pieces | 0.00 | 0.04 | 1.43 | 1.68 |
| Q12. Think about burning up calories when I exercise | -0.62 | 0.04 | 1.34 | 1.37 |
| Q20. Feel that others pressure me to eat | 1.03 | 0.06 | 1.37 | 1.24 |
| Q4. Have gone on eating binges where I feel that I may not be  able to stop | 0.63 | 0.05 | 1.31 | 1.21 |
| Q6. Aware of the calorie content of foods that I eat | -0.64 | 0.04 | 1.11 | 1.24 |
| Q24. Like my stomach to be empty | 0.55 | 0.05 | 1.19 | 1.09 |
| Q1. Am terrified about being overweight | -0.72 | 0.04 | 1.19 | 1.18 |
| Q18. Feel that food controls my life | 0.13 | 0.04 | 1.13 | 1.05 |
| Q3. Find myself preoccupied with food | -0.20 | 0.04 | 0.94 | 1.07 |
| Q14. Am preoccupied with the thought of having fat on my body | -0.18 | 0.04 | 1.04 | 1.00 |
| Q16. Avoid foods with sugar in them | 0.15 | 0.04 | 1.00 | 1.03 |
| Q7. Particularly avoid foods with a high carbohydrate content  (i.e., bread, rice, potatoes, etc.) | -0.06 | 0.04 | 0.97 | 1.02 |
| Q21. Give too much time and thought to food | -0.02 | 0.04 | 0.98 | 0.93 |
| Q2. Avoid eating when I am hungry | 0.40 | 0.05 | 0.83 | 0.94 |
| Q22. Feel uncomfortable after eating sweets | -0.01 | 0.04 | 0.83 | 0.81 |
| Q11. Am preoccupied with a desire to be thinner | -0.42 | 0.04 | 0.79 | 0.79 |
| Q17. Eat diet foods | -0.02 | 0.04 | 0.69 | 0.76 |
| Q10. Feel extremely guilty after eating | 0.15 | 0.04 | 0.75 | 0.67 |
| Q23. Engage in dieting behavior | -0.15 | 0.04 | 0.67 | 0.74 |
| Note. SE = standard errors; MNSQ = mean square residuals. | | | | |
